# Bibliometric analysis of literature on toxic epidermal necrolysis and Stevens-Johnson syndrome: 1940 – 2015

**DOI:** 10.1186/s13023-017-0566-8

**Published:** 2017-01-18

**Authors:** Waleed M. Sweileh

**Affiliations:** College of Medicine and Health Sciences, Department of Physiology and Pharmacology/Toxicology, An-Najah National University, Nablus, Palestine

**Keywords:** Stevens-Johnsons Syndrome, Toxic epidermal necrolysis, Bibliometric analysis

## Abstract

**Background:**

Stevens Johnson Syndrome (SJS) and toxic epidermal necrolysis (TEN) are rare but fatal adverse skin reactions that affect all age groups. In order to better understand literature on this topic, we conducted a bibliometric study using Scopus database to shed light on number and growth of publications, most active countries, institutions, journals and authors involved in publishing articles in this field, citation analysis, top cited articles, international collaboration, role of medications and genetic association. Bibliometric analysis will enrich the literature on these rare conditions and will provide baseline data for future comparison.

**Results:**

Three thousand eight hundred fifty-six journal articles were retrieved. The *h*-index of retrieved documents was 95. Growth rates of publications were highest from 1966 to 1975 and from 2006 to 2015. The United States of America (*n* = 640; 16.57%) was the leading country in number of publications. However, French and Japanese researchers and institutions were most active in publishing articles on SJS and TEN. International collaboration among active countries was relatively low and ranges from 32.5% for Swiss researchers and 1.47% for Spanish researchers. The most frequently mentioned medication in retrieved articles was carbamazepine (*n* = 146) followed by phenytoin (*n* = 114) and allopurinol (*n* = 112). Mycoplasma infection was mentioned in 111 articles. Most documents on SJS and TEN were published in dermatology journals, specifically *Archives of Dermatology*. However, in the last decade, top cited articles appeared in dermatology and pharmacogenetic journals. Carbamazepine was frequently encountered with Han Chinese and HLA-B 1502 terms while allopurinol was frequently encountered with HLA-B 5801 and Japanese terms.

**Conclusion:**

Bibliometric analysis reveals that research publications on SJS and TEN have been increasing since the l940s, with relatively low international collaboration. Documents are being published, not only in dermatology journals, but also in genetic, public health and general medicine journals. Research on SJS and TEN can be helpful to clinicians and researchers not only to document complications and fatal outcomes, but also to identify potential causative agents and potential ethnic variations to note gaps in research.

## Background

Stevens-Johnson syndrome (SJS) and toxic epidermal necrolysis (TEN) are uncommon, but serious and sometimes fatal adverse skin and mucous reactions [1]. Stevens and Johnson were the first to describe SJS in 1922 while Lyell was the first to describe TEN in 1956 [2, 3]. Up until early 1990s, erythema multiforme major (EMM) and SJS were considered similar clinical conditions. However, in 1993 EMM and SJS were identified as two distinct disorders [4]. TEN and SJS are now considered part of a continuum with SJS representing the less severe form of the reaction, and TEN representing the more severe end. The TEN involves larger total body surface area than SJS. The “SJS/TEN” is considered an intermediate form [5–7]. Incidence rates of TEN and SJS varies across the studies but it is generally less than two cases per million each year [7]. The reported mortality in patients with SJS varies from 3 to 10% while those with TEN have higher mortality rate and ranges from 20 to 40% [8]. The medical outcome of SJS and TEN varies depending on the medical condition of the patient when initiating therapy of the condition [9].

Both SJS and TEN are most often triggered by certain medications, most commonly anticonvulsants, non-steroidal anti-inflammatory drugs, and antibiotics [10, 11]. Unfortunately, a recent study showed that there is an under reporting of drug – induced SJS and TEN [12]. Common over-the-counter drugs such as paracetamol and ibuprofen have been reported in some cases of SJS and TEN [13]. According to Stevens Johnson Syndrome foundation (SJSF), any medication, including cocaine and Ginseng containing herbal products, can cause SJS and TEN [14]. Other causes of SJS and TEN include certain infections [15]. For unclear reasons, it was found that patients with human immune deficiency virus (HIV) have higher risk of developing SJS and TEN compared to un-infected people [7, 16–21]. Other infections such as *Mycoplasma pneumoniae* infections and herpes simplex viral infections were linked to SJS and TEN even without previous exposure to medications [22–27]. SJS and TEN are associated with certain HLA types [28–32]. The genetic basis of SJS and TEN might be responsible for variations in reporting of drug-induced SJS and TEN in different parts of the world [33]. Better understanding of these variation can help in developing preventive and therapeutic policies for SJS and TEN [34].

Being considered rare conditions, studies on SJS and TEN are required. Such studies are beneficial for pharmacologists, clinical toxicologists, and clinicians in various medical specialties such as dermatologists, pediatricians, and critical care specialists. One method to enrich literature about a certain topic is to provide detailed analysis of published literature on that particular topic. Such analysis of literature is called bibliometric analysis. Therefore, this study was carried out to give a bibliometric overview of literature on TEN and SJS. Bibliometric analysis is not intended to be a review article of scientific information on TEN and SJS, rather an analysis of published literature in terms of annual growth, productivity of various countries, institutions and authors, international collaboration, highly cited articles, and to lesser extent discussion of the content of highly cited articles. Bibliometric analysis on various medical subjects, including new subjects such as telemedicine have been carried out and published [35–44]. Such studies added new information to the scientific literature of these conditions and added momentum to research aspects of these conditions. Rare diseases and conditions should be given priority in bibliometric studies to establish baseline data for future comparison. Therefore, we believe that such a study will be a positive addition to the field of rare diseases in general and to SJS and TEN in particular.

## Methods

In bibliometric studies, literature on a certain topic need to be retrieved and analyzed as an initial step. To accomplish this, the largest database need to be used. Unfortunately, no single database is perfect. However, Scopus database is believed to be the largest and most accurate when compared with other databases [45, 46]. Therefore, for this study, Scopus was used and accessed through Hinari website.

The keywords entered in Scopus search engine were straight forward and consisted of “Toxic epidermal necrosis”, “Stevens-Johnson syndrome”, and “Lyell”s syndrome”. These keywords were used in title search separated by “OR” function. The time span of the study was determined from 1940 to 2015. The search query was limited to published journal articles with errata (corrections) documents being excluded. The search query looked like this in Scopus: (TITLE(“Toxic epidermal necrolysis”) OR TITLE(“Lyell”s syndrome”) OR TITLE(“Stevens-Johnson syndrome”) AND (EXCLUDE(PUBYEAR,2016)) AND (LIMIT-TO(SRCTYPE,“j”)) AND (EXCLUDE(DOCTYPE,“er”)).

One might question the validity of using title search instead of title-abstract-keyword approach. The use of title-abstract-keyword research will retrieve so many false positive results that will compromise the validity of retrieved data. However, the use of title search will minimize both false positive and false negative results. The validity of retrieved articles was based on manual check of the content of 10% of retrieved documents across all years and found to be free of false positive articles.

Retrieved articles can be analyzed from different aspects. Growth of publications was presented in decade interval. Growth rate was determine by calculating the difference in number of publications between two consecutive decades and dividing by the number of publications in the earlier decade. Scopus has the ability to rank countries, institutions and authors based on the number of articles they participated in. Articles with authors having different country affiliation are called multiple country publication (MCP) and considered to represent international collaboration of that country. On the other hand, articles in which all authors have the same country affiliation are called single country publication (SCP) and are considered to represent intra-country (within) collaboration.

Scopus provides researchers with the opportunity to do citation analysis. Retrieved documents can be sorted based on number of citations they received. Top cited articles are those that received the highest number of citations while articles with a minimum number of 100 citations are considered to be highly cited articles. Scopus provides total citations for retrieved articles and Hirsch index (*h*-index).

Visualization and mapping of specific results could be achieved through using a mapping program such as VOSviewer. In this study, data for countries, institutions, authors, and journals were presented in tables on the basis of a minimum contribution of 20 articles.

This study did not require approval of ethic committee and therefore, approval of study by medical ethics committee was not sought. Data presentation did not involve statistical analysis and Microsoft Excel program was used to accomplish most of the data presented in this study.

## Results

### General information

**Fig. 1:**
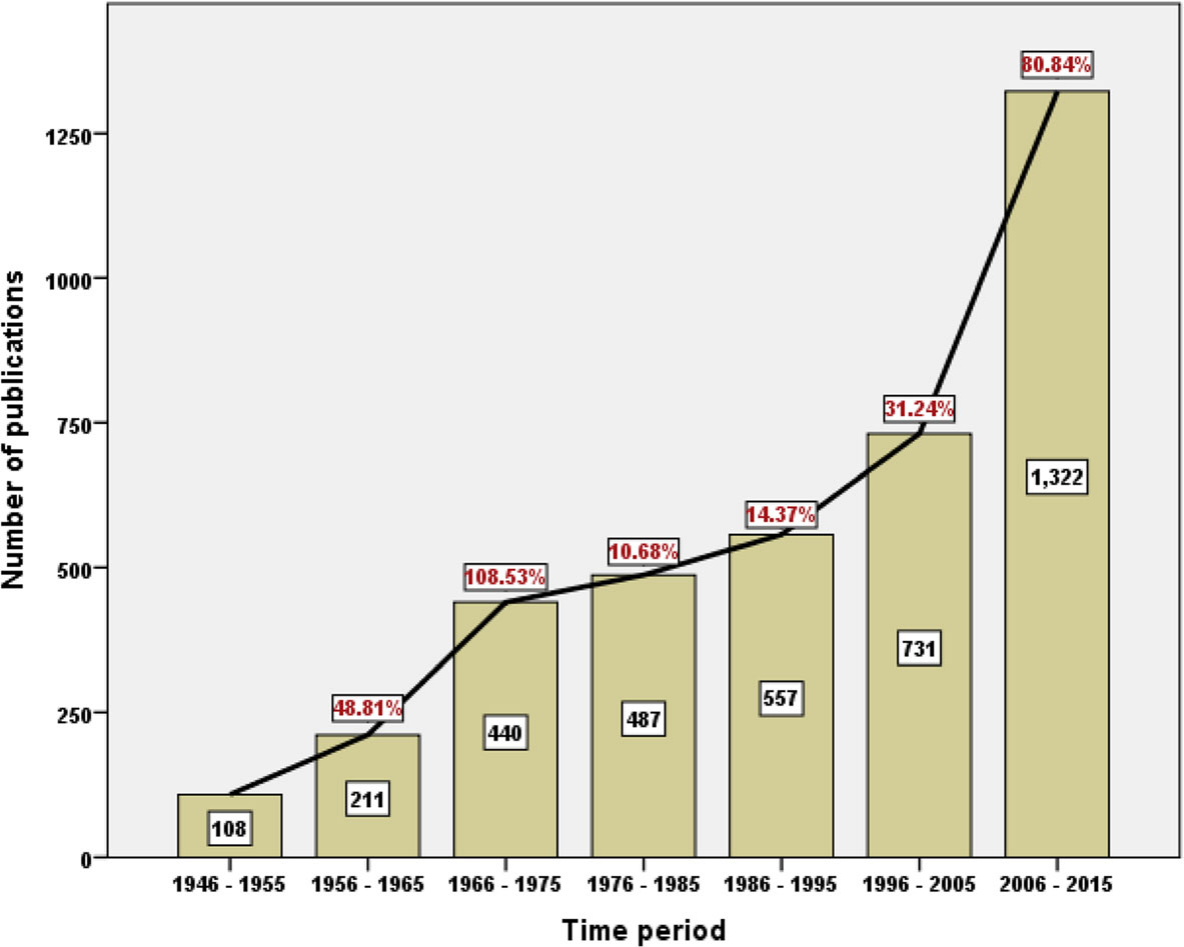
Growth of publications on SJS and TEN presented in decade intervals

**Fig. 2:**
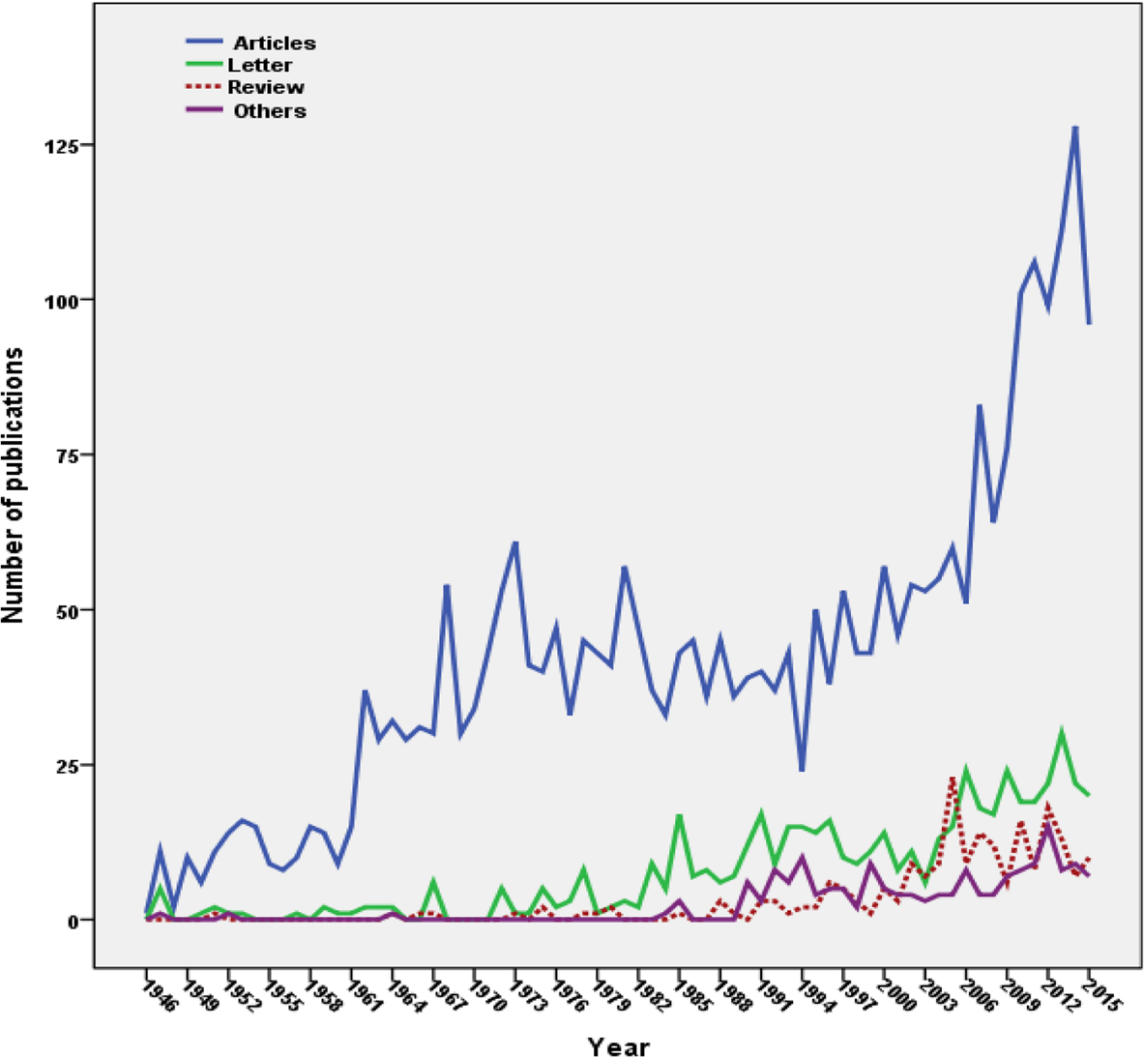
Annual growth of publications of various types of retrieved journal articles

Three thousand eight hundred fifty-six journal articles were retrieved. The majority of retrieved articles were research articles (*n* = 2948; 76.45%) followed by letters (*n* = 529; 13.72%) and reviews (*n* = 211; 5.47%) (Table 1). The majority of retrieved articled were written in English (*n* = 2,575; 66.78%). Other encountered languages are shown in Table 2. An article published in 1946 was the oldest record of SJS and TEN in Scopus database [47]. The growth of publications on SJS and TEN is presented in decade intervals in Fig. 1. The highest growth rates were seen during the periods from 1956 to 1965 and from 2006 to 2015. The annual growth of publication of various types of journal articles is shown in Fig. 2.

**Table 1:** Types of retrieved documents

Type of document	Frequency *N* = 3856	Percent
Article	2948	76.45
Letter	529	13.72
Review	211	5.47
Note	70	1.82
Conference Paper	51	1.32
Short Survey	25	0.65
Editorial	15	0.39
Article in Press	7	0.18

**Table 2:** Types of languages encountered in retrieved documents

Language	Frequency *N* = 3856	Percent
English	2575	66.78
French	231	5.99
Spanish	191	4.95
Russian	189	4.90
German	165	4.28
Italian	107	2.77
Japanese	91	2.36
Polish	79	2.05
Dutch	29	0.75
Korean	26	0.67
Portuguese	25	0.65
Chinese	19	0.49
Czech	19	0.49
Turkish	18	0.47
Hungarian	15	0.39
Norwegian	14	0.36
Romanian	14	0.36
Danish	13	0.34
Serbian	9	0.23
Swedish	7	0.18
Ukrainian	7	0.18
Hebrew	6	0.16
Slovak	5	0.13
Bulgarian	3	0.08
Croatian	3	0.08
Finnish	1	0.03
Greek	1	0.03
Undefined	24	0.62

*SJS* Stevens – Johnson Syndrome

*TEN* Toxic Epidermal Necrolysis

### Frequently encountered terms

**Fig. 3:**
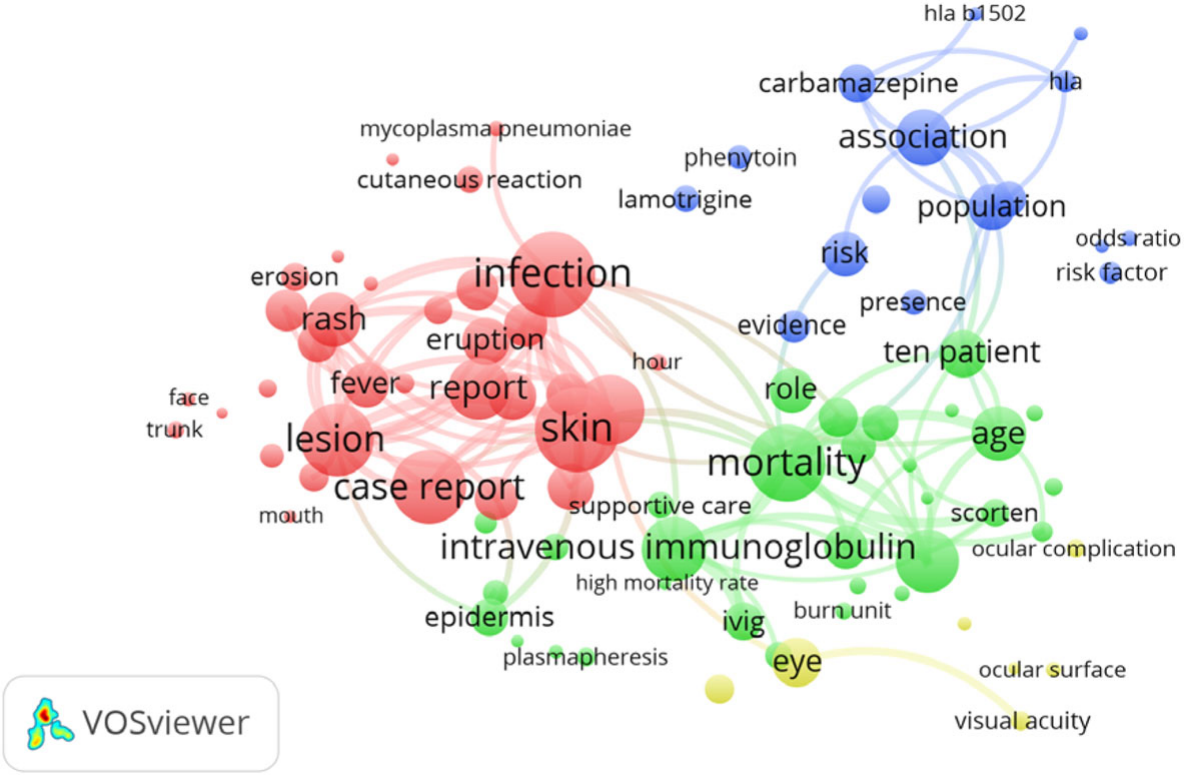
Network visualization of most frequently encountered terms

Network visualization of most frequently encountered terms is shown in Fig. 3. The map included four clusters: the first cluster (red) included terms that describes the SJS and TEN skin eruption and associated factors; the second cluster (green) included terms pertaining to treatment and potential outcome; the third cluster (blue) included terms pertaining to medications and genetic association with HLA types; and the fourth cluster (yellowish green) included terms pertaining to ophthalmic complications of SJS and TEN.

### Citation analysis

**Fig. 4:**
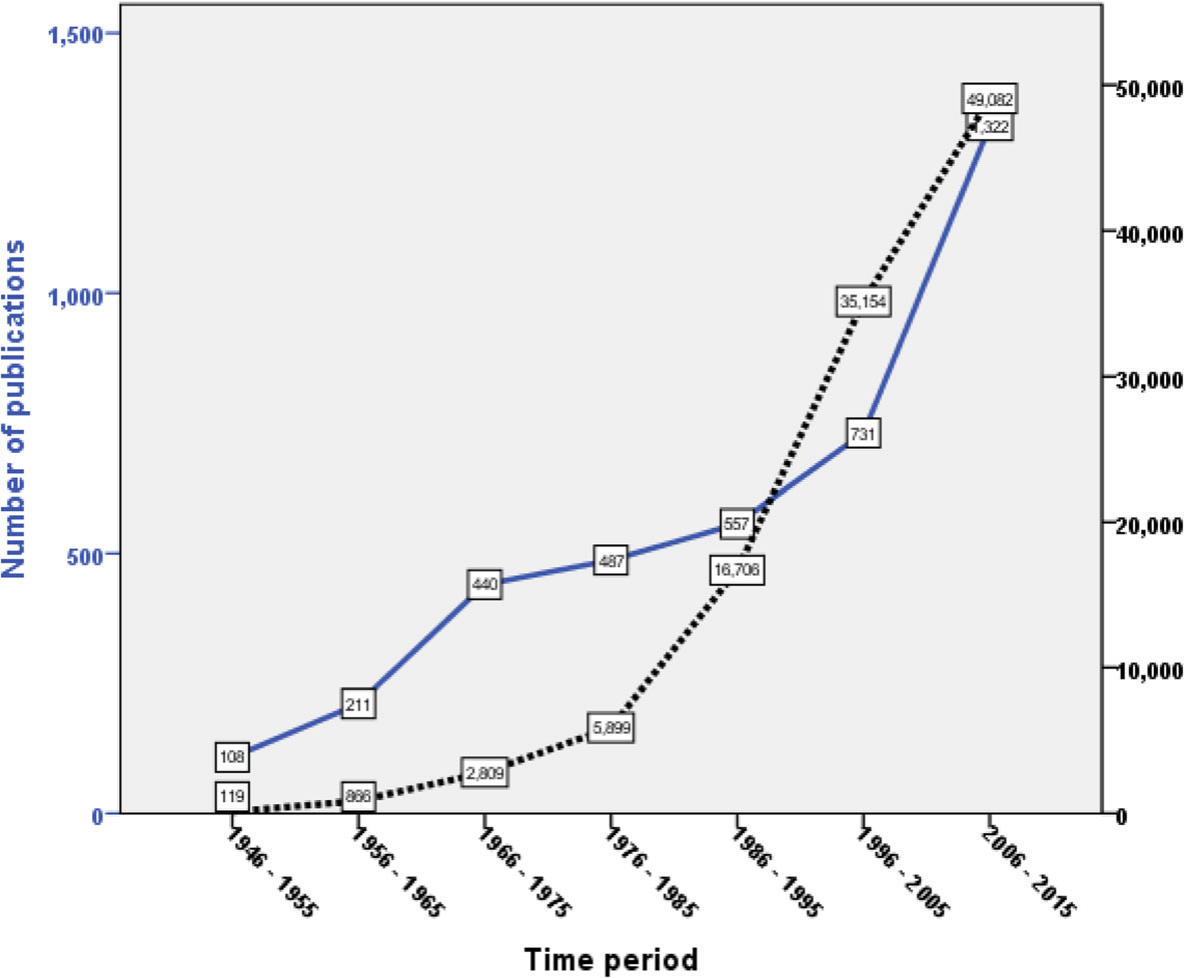
Growth of publications versus number of cumulative citations

Retrieved documents received a total of 4908 citations. The mean ± standard deviation of number of citations per article was 12.73 ± 41.57 while the median (Q1 – Q3) was 2 (0 – 10). The *h*-index of retrieved documents was 95. The cumulative number of citations showed a linear increase with time indicative of continuous interest, readability, and citations of SJS and TEN publications (Fig. 4). The review article entitled “*Medication use and the risk of Stevens-Johnson syndrome or toxic epidermal necrolysis*” [48] published in *New England Journal of Medicine* (*NEJM*) in 1995 received the highest number of citations (938). A total of 87 articles (2.25%) were highly cited.

### Top cited articles

Table 3 represents top cited articles across different time periods. Top cited articles during the period from 1946 to 1985 were mainly published in dermatology journals and the main theme of the top cited articles was description of SJS and TEN cases with emphasis on medication – induced skin reactions. During the period from 1986 to 2005, the top cited articles were published in dermatology journals and prestigious general science journals such as *Nature* and *Science*. The main theme of top cited articles during that period was classification of the condition, illness severity score, and molecular pathogenesis of the SJS and TEN skin reaction. During the time period from 2006 to 2015, top cited articles were published in pharmacogenetic journals as well as dermatology journals. The emphasis of top cited articles during that period was on the association between SJS. TEN, medications, and HLA typing.

**Table 3:** Top 10 cited articles on SJS and TEN in three different time periods

Rank	Top 10 cited articles for the time period 1946 – 1985 *N* = 1246		Top 10 cited articles for the time period 1986 - 2005 *N* = 1288		Top 10 cited articles for the time period 2006 – 2015 *N* = 1322	
	Reference	Number of citation	Reference	Number of citation	Reference	Number of citation
1	ᅟ	392	[72]	938	[73]	404
2	[74]	159	[75]	829	[76]	321
3	[77]	153	[78]	825	[79]	299
4	[80]	101	[81]	771	[82]	278
5	[83]	94	[84]	438	[49]	244
6	[85]	89	[86]	393	[87]	230
7	[88]	86	[89]	325	[90]	206
8	[91]	75	[92]	322	[93]	205
9	[94]	72	[95]	316	[96]	178
10	[97]	66	[98]	288	[99]	173
Main journals	7 Dermatology		6 Dermatology		4 Dermatology and 4 Genetic	
Main focus	Descriptive studies, ocular consequences, drug etiology		Illness severity Score, classification of TEN, and molecular aspects of therapy		Ethnic variations, role of genetics, and anti-epileptics induced TEN	

*SJS* Stevens – Johnson Syndrome

*TEN* Toxic Epidermal Necrolysis

### Active countries

**Fig. 5:**
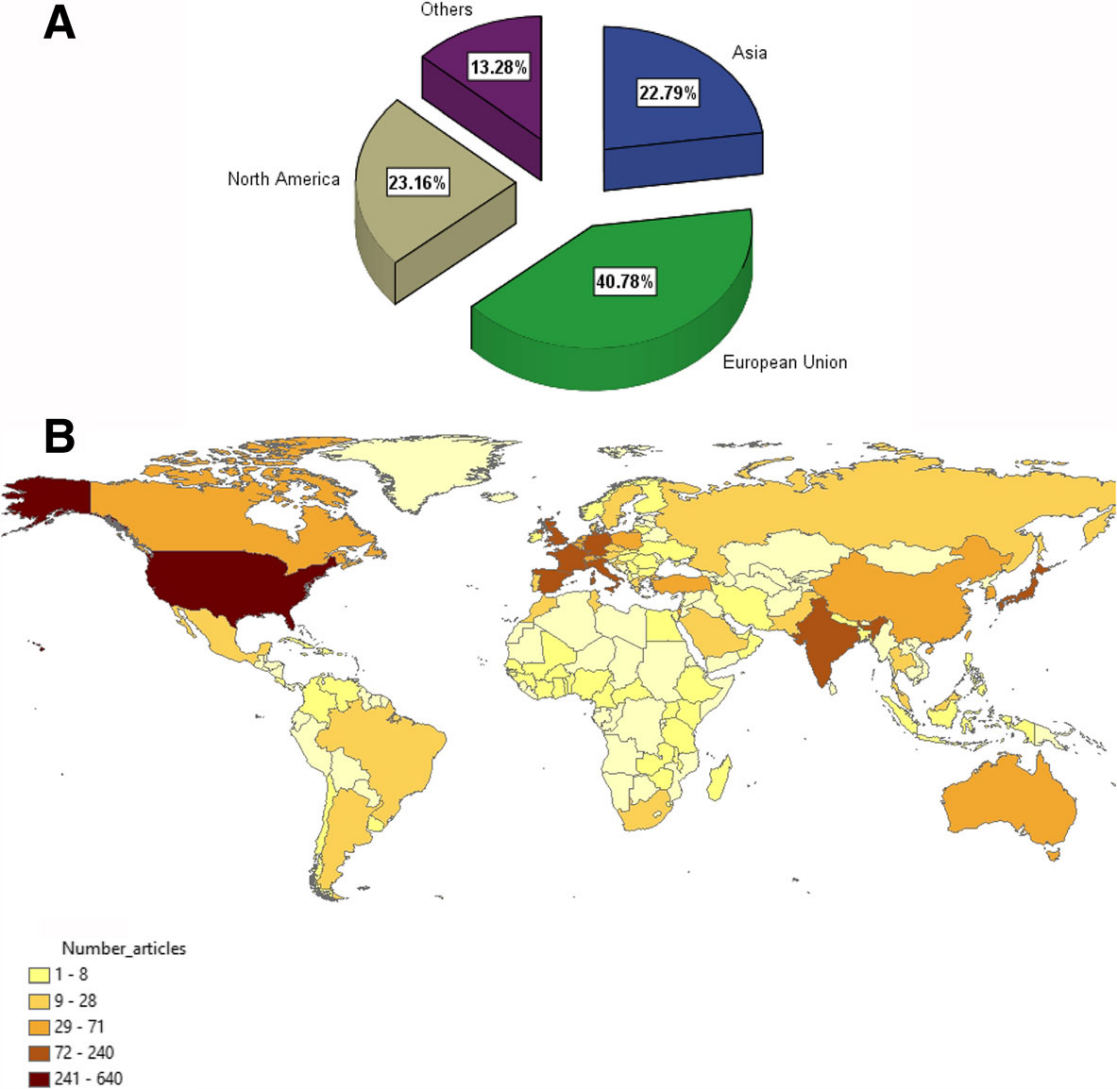
**a** Productivity of various world regions. **b** Geographic distribution of SJS and TEN publications using ArcMap 10.1 software

Researchers from 96 different countries contributed to the advancement of knowledge on SJS and TEN. Table 4 shows a list of active countries which had published a minimum of 20 publications on SJS and TEN. The total number of publications produced by these active countries was 2640 (68.46%). The United States of America (USA) (*n* = 640; 16.57%) ranked first in number of publications followed by Japan (*n* = 237; 6.14%), France (*n* = 234, 6.06%) and the United Kingdom (UK) (*n* = 199; 5.15%). When research productivity was stratified by world region, the European union had the greatest share followed by north America and Asia (Fig. 5a). Geographic distribution of research productivity is presented in Fig. 5b.

**Table 4:** List of active countries/territoris in publishing on SJS and TEN

Country/Territory	Frequency	% *N* = 3856	Number of collaborating countries	SCP	Percent	MCP	Percent
United States	640	16.60	36	572	89.38	68	10.63
Japan	237	6.15	7	225	94.94	12	5.06
France	234	6.07	19	193	82.48	41	17.52
United Kingdom	199	5.16	16	178	89.45	21	10.55
Germany	163	4.23	19	121	74.23	42	25.77
India	159	4.12	6	153	96.23	6	3.77
Italy	152	3.94	14	133	87.50	19	12.50
Spain	136	3.53	1	134	98.53	2	1.47
Turkey	71	1.84	3	68	95.77	3	4.23
South Korea	70	1.82	4	66	94.29	4	5.71
Taiwan	68	1.76	8	59	86.76	9	13.24
Canada	58	1.50	10	43	74.14	15	25.86
Belgium	54	1.40	4	49	90.74	5	9.26
Poland	50	1.30	4	49	98.00	1	2.00
Australia	46	1.19	4	43	93.48	3	6.52
China	45	1.17	4	37	82.22	8	17.78
Netherlands	44	1.14	8	33	75.00	11	25.00
Switzerland	40	1.04	9	27	67.50	13	32.50
Singapore	37	0.96	9	30	81.08	7	18.92
Israel	36	0.93	9	25	69.44	11	30.56
Brazil	28	0.73	7	22	78.57	6	21.43
Portugal	26	0.67	5	22	84.62	4	15.38
Austria	25	0.65	8	17	68.00	8	32.00
Mexico	22	0.57	4	18	81.82	4	18.18

*SJS* Stevens – Johnson Syndrome

*TEN* Toxic Epidermal Necrolysis

*SCP* Single Country Publication (intra-country publication)

*MCP* Multiple Country Publication (inter-country publication = International collaboration)

**Fig. 6:**
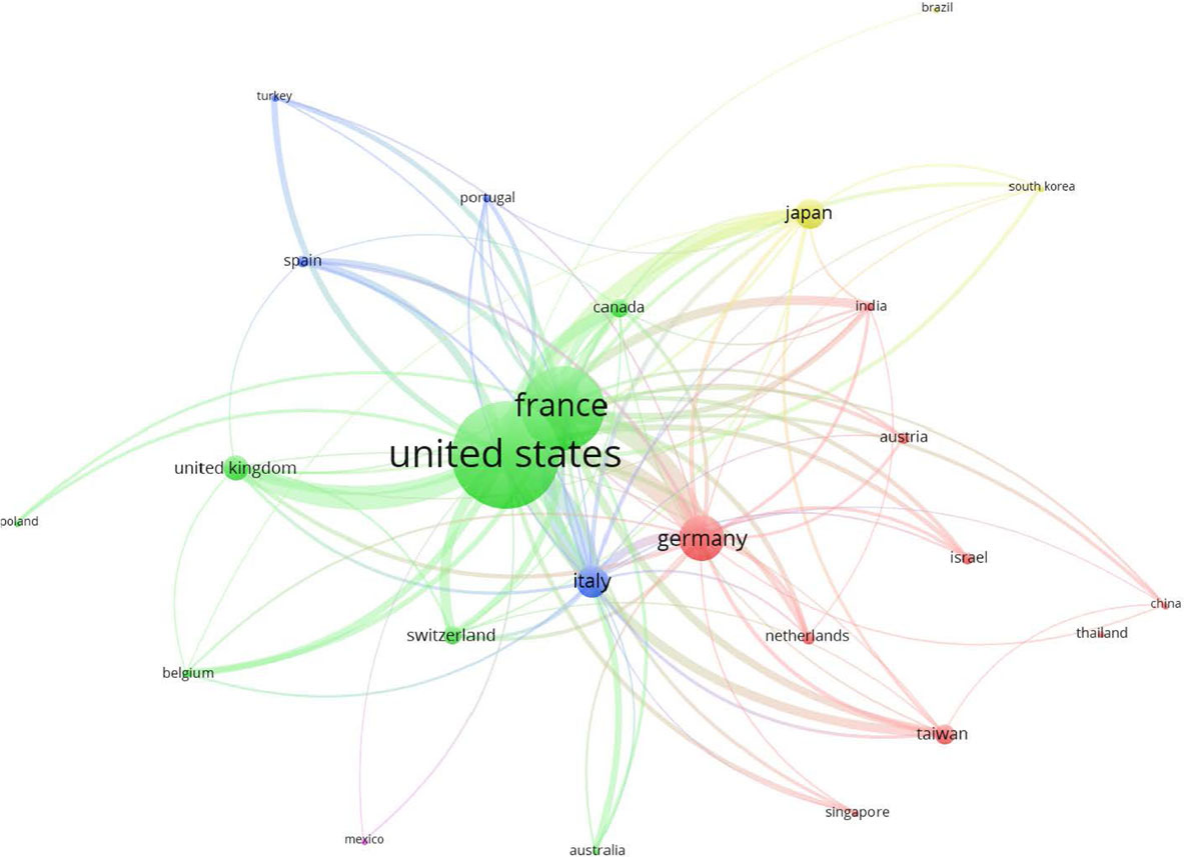
Network visualization map of citations for articles produced by active countries

Network visualization of citations received by articles produced by active countries is shown in Fig. 6. Publications form the USA received the highest number of citation followed by those from France, Germany, Italy and the UK. The volume of citations is correlated with size of the circle representing each country in network visualization map.

**Fig. 7:**
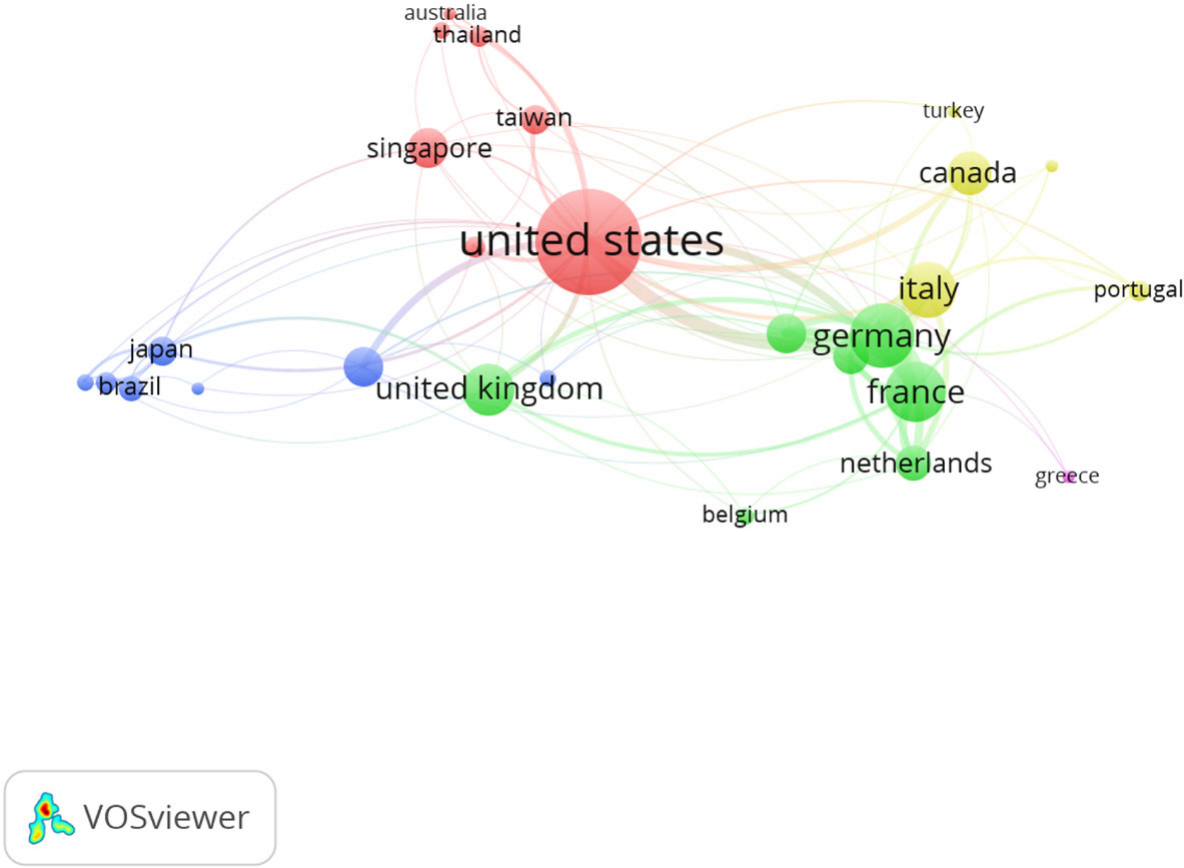
Network visualization map of country co-authorship (international collaboration)

International collaboration (MCP) was also assessed for the active countries. Approximately one third of articles (32.5%) produced by Switzerland had international authors. In contrast, only 1.47% of articles produced by Spain had lowest international authors. International collaboration among active countries is presented by network visualization map (Fig. 7). The thickness of the link between any two countries represents the strength of collaboration while the size of the circle assigned for that country represents the extent of international collaboration. The network visualization map showed that the strongest collaboration was between Germany and France. The USA had the largest circle size indicative of the highest extent of international collaboration. The percentage of MCP for the USA was not the highest. However, because of the very large number of published articles by the USA, the extent of international collaboration by the USA was the highest. When network visualization map was presented as a density visualization map, countries such as Germany, France, UK, Netherlands, Belgium, Israel, and Italy were found in a close cluster while the USA, China, Taiwan, Singapore, Thailand, and Australia were found in a second close cluster suggestive of close collaboration.

### Active institutions

**Fig. 8:**
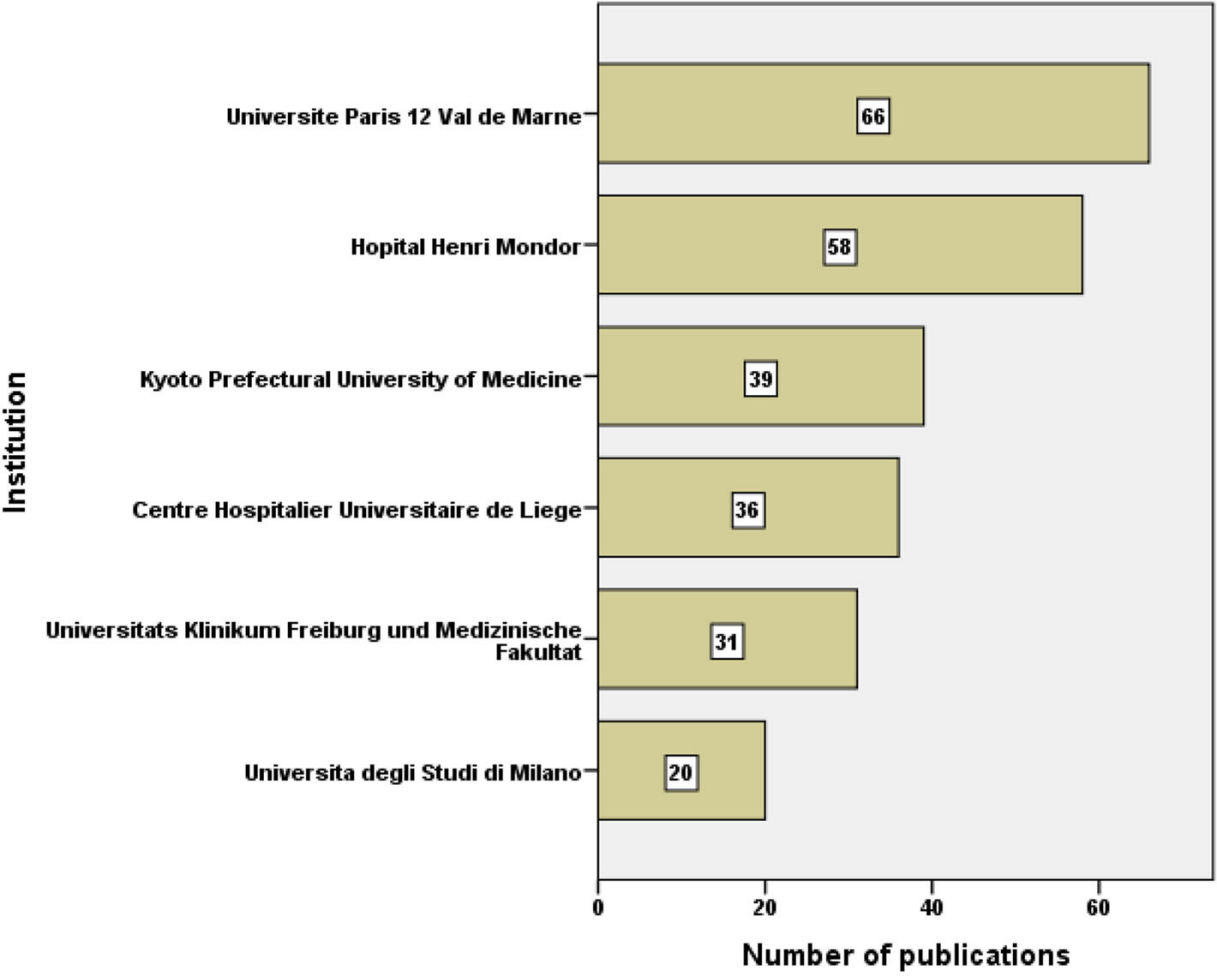
Active institutions in publishing about SJS and TEN

Active institutions in publishing articles on SJS and TEN were presented in Fig. 8. The most active institution was *Universite Paris 12 Val de Marne in France* (*n* = 66; 1.71%) followed by *Hospital Henri Mondor* in France (*n* = 58; 1.5%) and *Kyoto Prefectural University of Medicine* in Japan (*n* = 40; 1.03%). The first two active institutions were in France. When institutions with a minimum productivity of ten articles were analyzed for country/territory affiliation, the USA ranked first with eight institutions followed by Italy with six institutions, France with five institutions, Japan with five institutions, and Taiwan with four institutions.

### Active authors

**Fig. 9:**
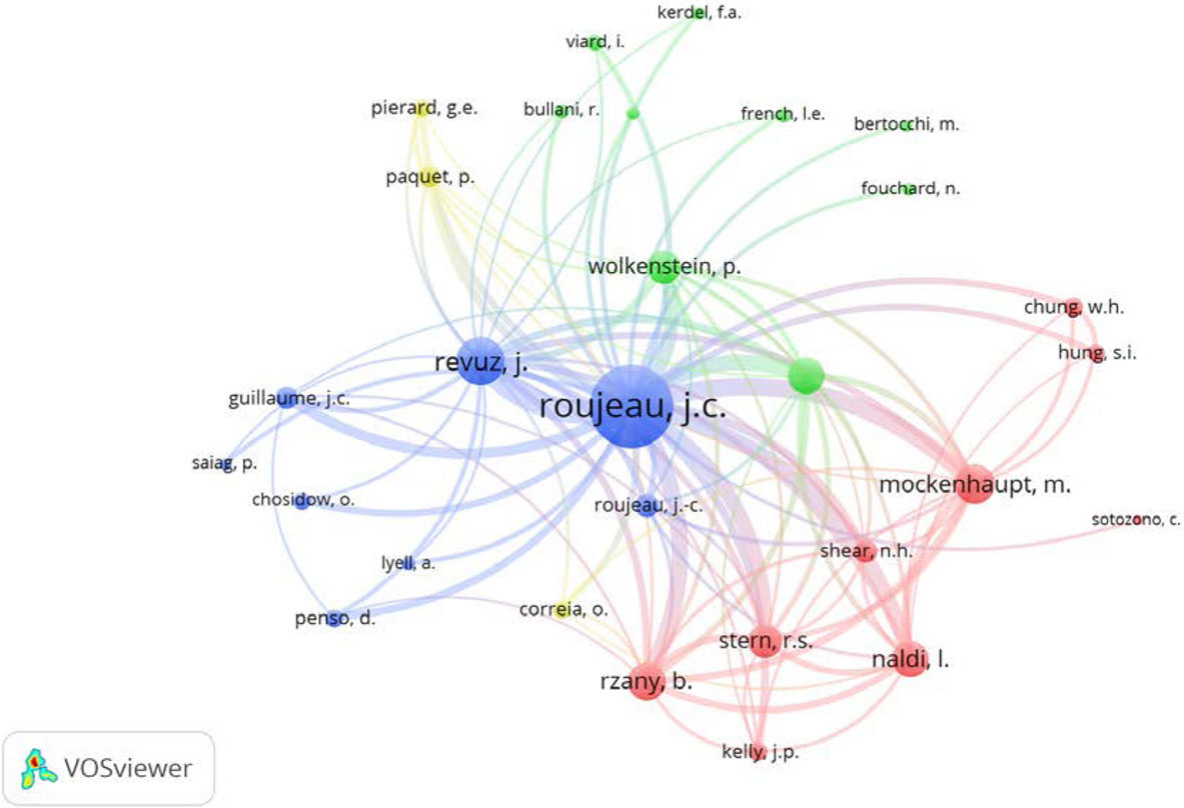
Co-citation analysis of active authors publishing articles on SJS and TEN

Active authors in publishing about SJS and TEN are shown in Table 5. Professor Roujeau, J.-C. from France had the highest contribution with 113 articles. Furthermore, Professor Roujeau, J.-C. had the highest *h*-index. Five active authors were French and three were Japanese. The other active authors were from Germany and Belgium. Co-citation analysis of active authors shows that Roujeau, J.-C was the most commonly co-cited author followed by Revuz, J. and Mockenhaupt, M (Fig. 9). Active authors were mainly from France, japan, and Belgium

**Table 5:** Active authors in publishing articles on SJS and TEN

Author	Frequency	% *N* = 3856	*h-*index	Country Affiliation
Roujeau, J.C.	113	2.93	48	France
Revuz, J.	57	1.48	28	France
Mockenhaupt, M.	51	1.32	23	Germany
Sotozono, C.	39	1.01	18	Japan
Paquet, P.	36	0.93	17	Belgium
Ueta, M.	30	0.78	13	Japan
Wolkenstein, P.	29	0.75	17	France
Aihara, M.	25	0.65	10	Japan
Touraine, R.	22	0.57	10	France
Piérard, G.E.	26	0.67	16	Belgium
Valeyrie-Allanore, L.	20	0.52	9	France

*h-index* Hirsch index

*SJS* Stevens – Johnson Syndrome

*TEN* Toxic Epidermal Necrolysis

### Active journals

**Fig. 10:**
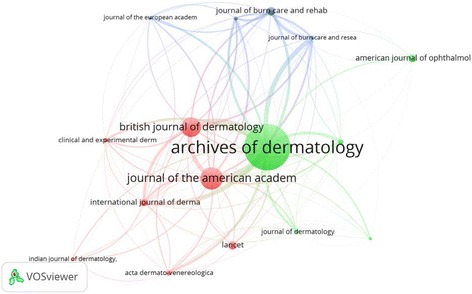
Citation analysis of active journals in publishing articles on SJS and TEN

Table 6 shows a list of 24 active journals involved in publishing articles on SJS and TEN. Each journal had published at least 20 articles on SJS and TEN. As expected, the majority of these journals were in the field of dermatology. Three journals were in the field of burns, two were in the field of ophthalmology and two were in the field of general medicine. Journal of *Archives of Dermatology* ranked first in number of publications followed by *British Journal of Dermatology* and *Lancet*. Citation analysis showed that articles on SJS and TEN published in *Archives of Dermatology* received the highest number of citations while those published in *Journal of The American Academy of Dermatology* and *British Journal of Dermatology* ranked second and third respectively (Fig. 10).

**Table 6:** Active journals in publishing articles on SJS and TEN

Journal	Frequency	% *N* = 3856	IF
Archives of Dermatology	123	3.19	0.14
British Journal of Dermatology	88	2.28	4.275
Lancet	83	2.15	45.217
Journal of The American Academy of Dermatology	80	2.07	4.449
International Journal of Dermatology	63	1.63	1.312
Clinical and Experimental Dermatology	46	1.19	1.315
Acta Dermato Venereologica	39	1.01	0.54
Journal of Dermatology	39	1.01	1.577
Annales De Dermatologie Et De Venereologie	36	0.93	0.07
Klinicheskaya Meditsina	33	0.86	0.08
Dermatology	32	0.83	1.569
Journal of Burn Care and Research	32	0.83	1.37
American Journal of Ophthalmology	30	0.78	3.831
British Medical Journal	28	0.73	19.967
Burns	28	0.73	0.89
Journal of The European Academy of Dermatology and Venereology	28	0.73	2.826
Korean Journal of Dermatology	27	0.70	0.04
Pediatric Dermatology	27	0.70	1.163
Actas Dermo Sifiliograficas	26	0.67	0.61
Annals of Pharmacotherapy	26	0.67	2.35
Journal of Burn Care and Rehabilitation	26	0.67	N/A
Indian Journal of Dermatology Venereology and Leprology	25	0.65	1.488
Cornea	21	0.54	1.18
Bulletin De La Societe Francaise De Dermatologie Et De Syphiligraphie	20	0.52	N/A

*IF* impact factor

*N/A* not applicable

*SJS* Stevens – Johnson Syndrome

*TEN* Toxic Epidermal Necrolysis

### Drug induced SJS and TEN, co-morbid conditions, and complications

The most commonly encountered medication in retrieved documents was carbamazepine which was mentioned in 146 documents followed by phenytoin which was mentioned in 114 and allopurinol which was mentioned in 112. Lamotrigene was mentioned in 93 documents while the antiviral nevirapine was mentioned in 53. Other mentioned medications include: valproic acid (*n* = 50), phenobarbital (*n* = 45), and sulfamethoxazole (*n* = 44). The over-the counter analgesic, paracetamol/acetaminophen was mentioned in 37 documents. Other causative agents include infections which include mycoplasma that had been mentioned in 111 documents. Other infectious agents include cytomegalovirus which was mentioned in seven documents. Vaccinations as a causative agent had been mentioned in 26 documents.

Human immune deficiency virus was co-mentioned in a total of 116 documents on SJS and TEN while cancer was co-mentioned in 91, tuberculosis in 29, hypertension in 19, and diabetes mellitus in 16 documents.

A total of 375 documents listed ocular/ophthalmic problems as complications of SJS/TEN, 26 mentioned urinary tract infections, and 95 mentioned sepsis/septicemia.

### Genetic predisposition

**Fig. 11:**
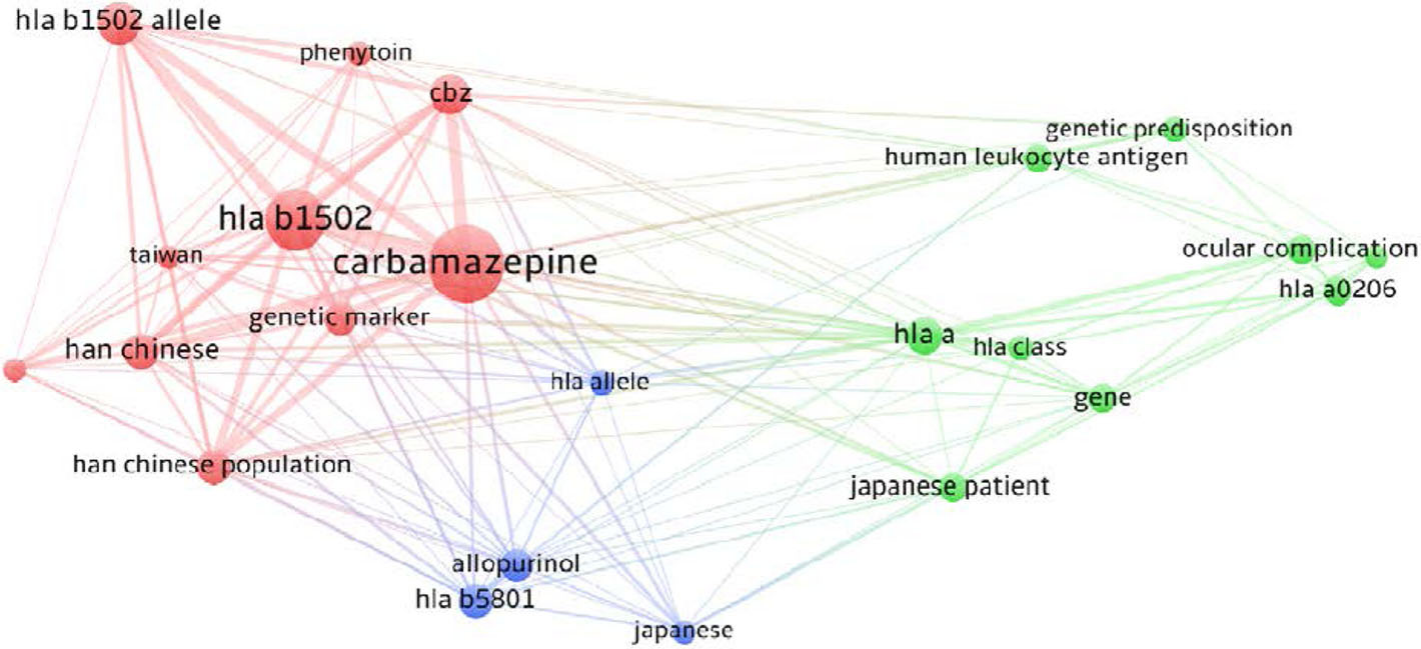
Network visualization map of predisposing genetic factors to SJS and TEN

A total of 65 documents discussed the genetic risk factors for developing SJS and TEN. Visualization map for terms present in title/abstract of these documents is shown in Fig. 11. The map consisted of three cluster (red, green and blue). Each cluster represents terms that were commonly mentioned together. Cluster number one included the following items: (carbamazepine/CBZ, genetic marker, Han Chinese/Han Chinese population, HLA b allele, HLA b 1502/HLA b 1502 allele, phenytoin, and Taiwan. The second cluster included the following items: (gene, genetic predisposition, HLA-A, HLA-A0206, HLA class, Human Leukocyte Antigen, Japanese patients, and Ocular complications. The third cluster included the following terms: (Allopurinol, HLA allele, HLA-B 5801, and Japanese).

## Discussion

In this study, we presented a bibliometric overview of literature on SJS and TEN published in the past seven decades. SJS and TEN are associated with high mortality rate and medications play a role as causative agents of SJS and TEN. Therefore, SJS and TEN are conditions with multidisciplinary aspects from a research and clinical management points of view [49, 50]. Our results showed that international collaboration in research pertaining to SJS and TEN is relatively low when compared with other fields. This might be due to the clinical nature and time course of the conditions. SJS and TEN are not chronic condition and except for the genetic component of these conditions, little can be done for international collaboration in research on SJS and TEN.

Our study showed that number of publications on SJS and TEN increased slowly and gradually over the past half century. This increase might be due to natural growth of medical publications in the past half century or due to increased reported cases of drug-induced SJS and TEN in the past half century. No doubt that the increasing number of newly approved drugs could be associated with new cases of SJS and TEN. Another potential explanation for the increased number of publications on SJS and TEN is the potential association of some infectious diseases such as *Mycoplasma pneumonia*, HIV, hepatitis, Cytomegalovirus, and others with SJS and TEN.

Our study showed that only two thirds of retrieved articles were in English. This suggests that cases of SJS and TEN are found and reported by non-English language from all over the world. Results of our study indicated that countries in northern America, Latin America, Europe, Middle East, and Southeast Asia were among active countries in publishing on SJS and TEN. Together with the high value of *h*-index of retrieved documents, it can be deduced that SJS and TEN are considered a serious and important research topic with high number of readers. Furthermore, the fact that some journals in the active list have high IF is suggestive of the clinical importance of SJS and TEN as a research and medical topic.

Our finding that the USA had the largest share of publications was not surprising given the huge budget allocated for research and the vast number of research centers in the USA. Several bibliometric studies on various medical topics concluded that the USA ranked first in research productivity [39, 40, 51–53]. However, it was surprising that most active authors were either French or Japanese and none were Americans. This could be attributed to regional differences in commonly prescribed drugs or due to differences in genetic predisposition [54, 55]. The analysis of Japanese Adverse Drug Event Report (JADER) database and analysis of the time-to-onset profile of SJS and TEN yielded data that are different from the Europeans [54]. A similar study was the EuroSCAR-study which is a multicenter European study carried out to evaluate medication induced severe cutaneous adverse reactions (SCAR) [55]. The EuroSCAR registry classified drugs that induce SJS/TEN into high, moderate, and low risk drugs with allopurinol and carbamazepine being in the high-risk category [56].

Our study showed that anti-epileptic drugs, particularly carbamazepine, phenytoin, lamotrigene and others were most commonly encountered in SJS and TEN literature. Different studies reported different main causative drugs of SJS and TEN possibly due to ethnic and genetic variations or due to different types of prescribing pattern. An Indian multicentric retrospective study of drug – induced SJS and TEN found that antimicrobials, nonsteroidal anti-inflammatory drugs, and antiseizure drugs were the most commonly associated groups with SJS and TEN and that antiseizure drugs were more often associated with TEN than other drugs [57]. A 20-year study in children in tertiary care hospital in Thailand found that antiepileptic drug group was the most common causative drug, followed by antibiotic drug group, and others which included nonsteroidal antiinflammtory drugs (NSAIDs) and chemotherapy drugs [10]. A study in Indonesia found that most common causative drugs were paracetamol, carbamazepine, amoxicillin, ibuprofen, rifampicin, and trihexyphenidyl [58].

Our study showed and emphasized previous findings of associations between certain genes and drug – induced SJS/TEN. For example, SJS/TEN induced by anti-epileptics such as carbamazepine and phenytoin is associated with HLA-B*15:02 in Han Chinese people [59]. Association of HLA-A 31:01 with carbamazepine – induced SJS have been reported in children [60]. A study in Thailand concluded that there is an association between HLAB (*) 58:01 and allopurinol-induced SJS-TEN in a Thai population [61]. Similar findings were obtained in Korean cases [62]. Presence of sever ocular complications in SJS/TEN have been linked to certain HLA types such as HLA-A*02:06 [63].

The top cited articles on SJS and TEN discussed several important issues in the field including ones on medication – induced SJS and TEN, genetic and ethnic role, illness severity scoring, treatment potential and others. The role of immunoglobulin in treatment of SJS/TEN is one of the top cited issues on SJS and TEN. Intravenous immunoglobulin as a therapeutic option has shown efficacy in reducing length of skin reactions and reducing severity of symptoms [64–66]. Recent published guidelines for the management of SJS and TEN included the removal of offending cause plus the initiation of high dose parenteral corticosteroids [67]. Use of plasmapheresis as an adjuvant therapy in SJS and TEN have shown beneficial effects [68–71].

Bibliometric studies are not without limitations. Despite that Scopus is the largest database available, articles published in non-indexed journals were not included in this study. Furthermore, the interchangeable use of SJS and EMM in the 1980s made the accuracy of results obtained during that period less than ideal. The keywords were used in title search which might have caused some data loss. However, title search is important to maintain high level accuracy and minimize false positive results. Scopus database retrieved not only countries but also distinct territories and that is why China and Taiwan appeared as separate countries/territories in genetic map despite that both are of similar ethnic group because Scopus database has China and Taiwan as separate entities/territories. Finally, the use of Web of Science (WoS) is believed to give data published in journals with impact factor as reported by Journal Citation Report. Therefore, WoS is believed to retrieve data in influential journals. In this manuscript we sought to cover the largest number of publications and therefore we did not utilize WoS.

## Conclusion

Our study showed that there was a gradual growth of publications on SJS and TEN in the past six decades. Bibliometric analysis of literature on SJS and The USA is important for clinicians and research to pinpoint gaps and understand worldwide aspect of these conditions regarding differential epidemiology and ethnic variations. Young researchers will learn from such bibliometric analysis studies about top cited articles on the conditions and names of institutions and authors who are leaders in the subject. Mapping genetic background and ethnicities is also of benefit for understanding medication – induced SJS and TEN among various ethnic groups. Despite the importance of the topic, the extent of international collaboration on the topic was relatively low, something which needs to addressed and encouraged.

## Abbreviations

SJS: Stevens – Johnson Syndrome
TEN: Toxic epidermal necrolysis
